# Crime of “Burking”

**Published:** 1875-09

**Authors:** 


THE CRIME OF "BURKING."—On the 29th of November, 1827, an old man by the name of Donald died in West Port, one of the purlieus of Edinburg. He lodged with an Irishman named William Hare, and died owing him four pounds. His creditor saw but one way of reimbursing himself, and that was by disposing of the old man's body to the doctors. Hare found a ready accomplice in William Burke, another Irishman, and also one of his lodgers. The body was removed from the coffin, and a bag of tanner's bark substituted for it. The lid was screwed down and the little funeral went off as usual. The same evening, Hare and Burke stealthily repaired to the university, and, meeting a student in the yard, asked for the rooms of Dr. Monroe, the Professor of Anatomy. The student happened to be a pupil of Knox's, and, upon discovering their errand, he advised them to try Knox's place in Surgeon's Square. There they sold the body for £7 10s., a large sum for them, and very easily obtained. They had not courage to go into the regular business of body-stealing, and so Hare, the vilest of the two, suggested a fresh stroke of business, which was to inveigle the old and infirm into his quarters and "do for them." Hare started in search of a victim; and, prowling through the slums, met an old woman half drunk, and asked her to his house. He gave her whisky until she became comatose, and then with Burke's assistance strangled her. The body brought £10.

The appetite of the vampires was now sharply whetted, and they entered systematically upon the work of murder. Vagrants, street-walkers, and imbeciles were allured on various pretexts to the house of Hare, made dead drunk, and suffocated. Emboldened by their success, they began to pursue their thuggish practices even in daylight. A woman named Docherty was stifled, and her body left half-exposed under some straw was seen by two lodgers, who notified the police. Thirteen victims had been secured in eleven months, and all taken to the same place and sold. The prisoners were tried December 24, 1828, when Hare, the blackest of the villains, was let off by turning "state's evidence," and Burke was convicted, hanged and dissected.

The effect produced upon the public by this horrible disclosure is indescribable. A new and unheard-of crime, that of "Burking," was added to the list of atrocities of which human fiends are capable. Astonishment and terror spread through the community. Households gathered their members within doors before dusk; workmen walked home from their night's toil in groups, as if in fear of being waylaid. The facts were appalling enough; but a thousand exaggerations and inventions filled the air, and intensified the universal excitement.—*From "A Popular Verdict," in Popular Science Monthly for September.*

NOTE.—We have heard the above story a little differently related by a well-informed physician of Edinburgh. It was a waife of the Canon Gate, an inoffensive and half-witted boy, generally known over the city by the nick-name "Doffy Jim," who was decoyed into the obscure, cheap lodging house of Hare and "Burked." The medical student above referred to was the prosector and anatomical assistant of Knox, and was none other than the present illustrious surgeon of Great Britain, Sir William Ferguson. So well known was Doffy Jim, it was deemed prudent to remove the head from the cadaver and consign it to the "pit," where it was subsequently found. This circumstance, so unusual, at once excited remark in Knox's lecture room, and was soon coupled with the other observed fact of the sudden and unaccounted for disappearance of Doffy Jim from the streets where he had for years made himself useful as an errand boy. Through Ferguson, who received the body, it was traced back to Burke and Hare. The former was executed, and his skeleton, with that of Doffy Jim, is still to be seen in the Museum of the University at Edinburgh. In a manner scarcely satisfactory at this day to the people of Edinburgh, Knox was driven forth under the lash of popular indignation, and died a miserable and friendless wanderer in the earth, whilst Ferguson found refuge in London, and has reaped the highest honors of the great metropolis.

